# Effect of situational simulation teaching combined with the AIDET framework on communication skills training for oncology residents

**DOI:** 10.1007/s00520-025-09570-y

**Published:** 2025-05-28

**Authors:** Yao Liang, Zhuo Wei Liu, Hai Bo Qiu, Jian Hong Peng, Yong Hong Li, Tang Li, Wan Huang, Wan Fei Xie, Hui Yan Luo, Ming Kuang

**Affiliations:** 1 State Key Laboratory of Oncology in South China, Guangdong Provincial Clinical Research Center for Cancer, Guangzhou, China; 2 Department of Gastric Surgery, Sun Yat-Sen University Cancer Center, Guangzhou, China; 3 Department of Urinary Surgery, Sun Yat-Sen University Cancer Center, Guangzhou, China; 4 Department of Colorectal Surgery, Sun Yat-Sen University Cancer Center, Guangzhou, China; 5 Clinical Skills Training Center, Sun Yat-Sen University Cancer Center, Guangzhou, China; 6 Department of Science and Education, Sun Yat-Sen University Cancer Center, Guangzhou, China; 7 Department of Medical Oncology, Sun Yat-Sen University Cancer Center, Guangzhou, China; 8 Zhong-shan School of Medicine, Sun Yat-Sen University, Guangzhou, China

**Keywords:** AIDET framework, Situational simulation teaching, Oncology residents, Communication skills

## Abstract

**Purpose:**

This study aims to develop and implement a situational simulation teaching model based on the AIDET communication framework (SST-AIDET) for oncology residents and evaluate its effectiveness in enhancing communication skills.

**Methods:**

A total of 117 third-year postgraduate residents at Sun Yat-sen University Cancer Center (SYSUCC) were randomly assigned to either a control group (*n* = 63) receiving traditional teacher-centered instruction or an intervention group (*n* = 54) undergoing SST-AIDET training. The intervention included theoretical instruction, simulation-based teaching, and discussion sessions. Communication skills were assessed through an Objective Structured Clinical Examination (OSCE), using the SEGUE Framework, the Chinese Physician–Patient Communication Assessment Scale (CPPC-AS), and the Consultation and Relational Empathy (CARE) measure.

**Results:**

The intervention group achieved significantly higher median scores in the SEGUE Framework (22 vs. 18, *P* < 0.001), CPPC-AS (with superior task, performance, and total scores, all *P* < 0.001), and CARE measure (45 vs. 43, *P* < 0.001) compared to the control group. A greater proportion of residents in the intervention group attained “excellent” scores in SEGUE and CARE assessments.

**Conclusions:**

The SST-AIDET teaching model significantly enhances communication skills among oncology residents and may improve standardized patient satisfaction, which is worth actively promoting in clinical teaching work.

**Supplementary Information:**

The online version contains supplementary material available at 10.1007/s00520-025-09570-y.

## Introduction

Effective communication is a cornerstone of clinical practice and is recognized globally as a critical competency in medical education. The Global Minimum Essential Requirements in Medical Education (GMER) [1] and the Accreditation Council for Graduate Medical Education (ACGME) [2] both emphasize its importance, identifying communication as a core skill required for resident physician training. Doctor-patient communication encompasses sharing information, building collaborative relationships with patients and their families, coordinating with healthcare teams, and delivering difficult news. Research consistently highlights the positive impacts of effective communication, including improved patient attitudes and expectations, enhanced satisfaction, reduced anxiety, better psychosocial well-being, increased treatment adherence, and improved clinical outcomes [3, 4].

The AIDET communication model, developed by the Studer Group, offers a structured approach to patient interactions through its five steps: Acknowledge, Introduction, Duration, Explanation, and Thank [5]. This framework standardizes language and procedures, enabling medical professionals to communicate with clarity and precision. By guiding interactions with clear and courteous language, the AIDET model improves patient understanding of medical conditions and expectations, reduces patient anxiety, enhances compliance and satisfaction, and fosters confidence among healthcare staff [6–8]. Moreover, its widespread application in training programs for clinical staff, nursing, and pharmacy students underscores its value in bridging theoretical communication concepts with practical skills [9–11] and has become a valuable tool for advancing doctor-patient communication [12]. Additionally, situational simulation teaching, which incorporates role-playing, object demonstration, and hands-on practice, creates immersive learning environments that align with specific educational objectives [13]. This method promotes active engagement, strengthens teamwork, and improves teaching efficacy [14].

This study seeks to develop and implement a Situational Simulation Teaching model based on the AIDET framework (SST-AIDET) to train oncology residents in communication skills and to evaluate its effectiveness.

## Method

### Participants

**Fig. 1:**
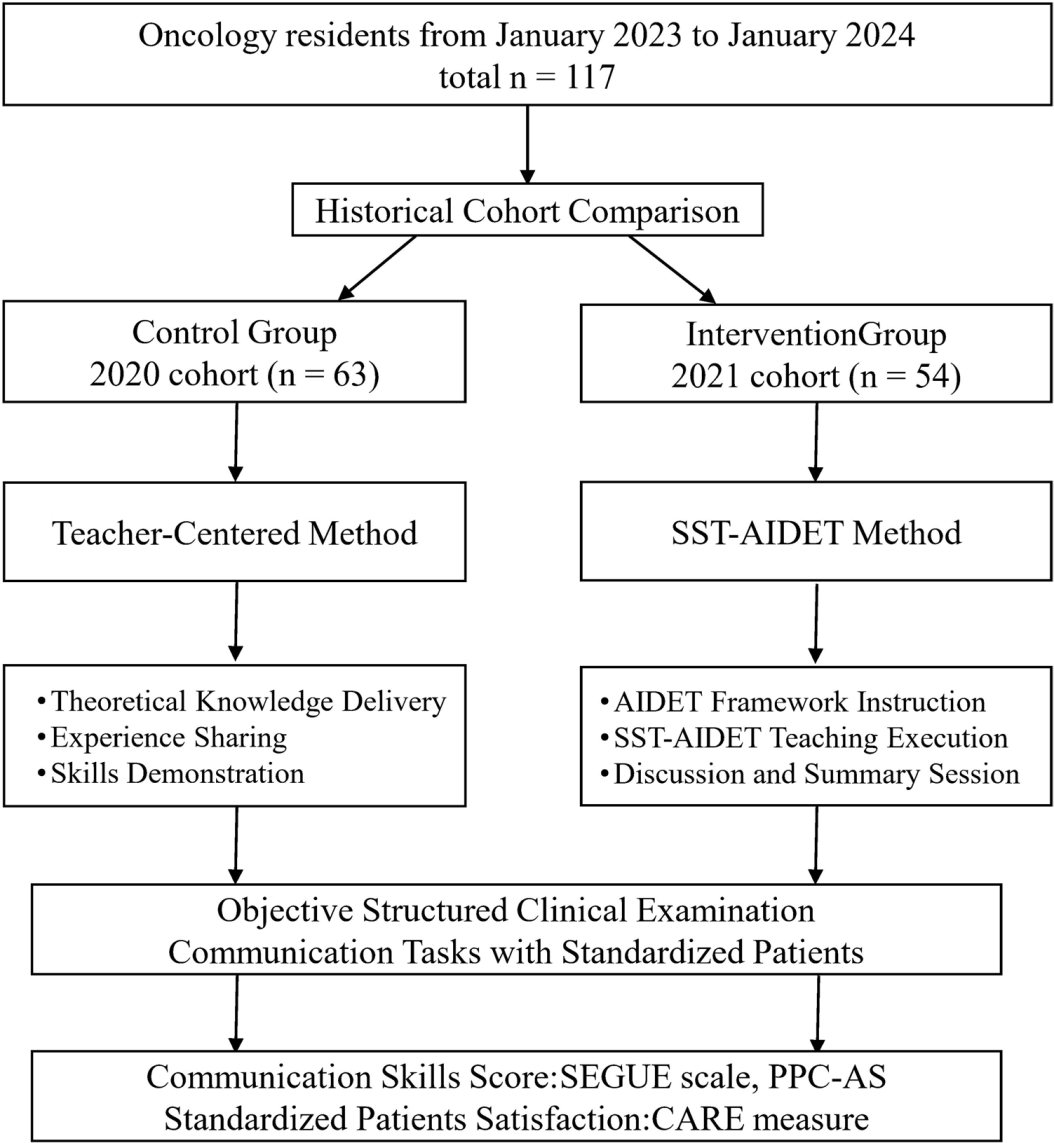
Flow diagram of design and procedure

The study included 117 third-year postgraduate residents from Sun Yat-sen University Cancer Center (SYSUCC), enrolled between January 2023 and January 2024, none of whom had prior experience in doctor-patient communication courses. A historical cohort design was adopted to minimize cross-cohort contamination between training groups. Sixty-three residents (53.8%) from the 2020 cohort were assigned to the control cohort and received traditional teacher-centered instruction. Meanwhile, 54 residents (46.2%) from the 2021 cohort were assigned to the intervention group and underwent in SST-AIDET communication training (Fig. 1).

### Study design

The two groups were further divided into smaller units, each comprising six to nine students, and all 117 students were taught by the same instructors.

#### Traditional teacher-centered method

The control group received traditional communication training without structured frameworks (e.g., AIDET) or simulated scenarios. Lectures delivered theoretical knowledge on communication, supported by slides, videos, and case analyses. Instructors shared personal experiences; demonstrated basic skills for medical history-taking, patient inquiries, and updates; and conducted live demonstrations with inpatients and their families. Students observed these interactions. The total instructional time was 3 h.

#### SST-AIDET method

The SST-AIDET method, conducted over four sessions within 3 h, was employed for the intervention group as follows:

1. AIDET framework instruction: Participants were introduced to the theoretical foundations of the AIDET communication model, focusing on its five core elements (Supplementary Table S1). This session aimed to build understanding of the AIDET framework’s concepts and procedures.
2. SST-AIDET teaching execution: Instructors selected four realistic doctor-patient communication scenarios from SYSUCC and developed scripts using the AIDET framework (Supplementary Table S2). Each simulation involved three roles—doctor, standardized patient (SP), and family member-played by the participants, while others observed. SPs had undegone a 4-h training program led by experienced clinicians and communication specialists. The instructor introduced the scenario, assigned roles, and distributed role-specific scripts. SPs received scripts detailing patient information and communication cues, doctors were given scenarios and brief patient profiles, and observers received all scripts for evaluation purposes. After a 15-min preparation, participants performed script-based simulations. The SPs enacted emotions and reactions specified in their scripts, while doctors crafted communication responses guided by the AIDET model, incorporating observations of patient and family member expressions. Observers documented the performance. Roles rotated after each simulation.
3. Discussion and summary session: Following each simulation, the instructor facilitated peer evaluations and reflections, providing corrective feedback on observed doctor-patient interactions. Any issues that were identified were addressed by demonstrating improved communication techniques.

### Evaluation

Residents’ doctor-patient communication skills were evaluated using the Objective Structured Clinical Examination (OSCE) in June 2023 and June 2024. A highly skilled attending physician served as the SP during these assessments. Examinees were randomly assigned scenarios that required them to perform tasks such as delivering bad news, obtaining informed consent, conducting preoperative discussions, and providing discharge instructions. Each OSCE session lasted 14 min.

To ensure objectivity, two independent supervising experts, each with over 10 years of teaching experience and no involvement in the course, along with the SP, assessed the residents’ communication skills on-site. These evaluators were blinded to the residents’ SST-AIDET training status to minimize potential bias. The final assessment score was determined by averaging the evaluations of the two experts.

#### Doctor‐patient communication skills

The SEGUE Framework, developed and validated by Makoul, was used to assess the residents’ communication skills [15]. This framework includes 25 items, grouped into five dimensions: “Set the Stage” (5 items), “Elicit Information” (10 items), “Give Information” (4 items), “Understand the Patient’s Perspective” (4 items), and “End the Encounter” (2 items). Each item is scored 1 for “yes” and 0 for “no,” with a maximum of 25 points. Grading standards are as follows: excellent (≥ 23 points), good (20–22 points), passing (15–19 points), and failing (< 15 points). The scoring system awards points for communication content (items 1–4, 6–11, 16–18, 20–21, 24–25) if the corresponding behavior is observed at least once. For communication technique items (items 5, 12–15, 19, 22–23), points are deducted if any required actions are omitted. A higher score reflects stronger communication skills.

The Chinese Physician–Patient Communication Assessment Scale (CPPC-AS), developed by the Department of Science and Education of the National Health Commission of China (Supplementary Table S3), is used as a grading standard in the national final examination for resident doctors in China. This scale includes two dimensions: Task (8 items, each worth 5 points) and Comprehensive Performance (10 items, each worth 6 points), for a total of 100 points.

#### Standardized patient satisfaction

The Consultation and Relational Empathy (CARE) measure is a tool designed to evaluate the quality of communication and empathy demonstrated by residents during patient encounters [16]. It consists of 10 items with specific explanations for understanding. Each item is scored on a scale from poor to excellent (1–5), with a total score ranging from 10 to 50 points. Utilized by the SPs to assess residents based on personal experiences and feelings, a higher CARE score indicates greater satisfaction from the SP’s perspective.

### Statistical analysis

Categorical variables are presented as counts with corresponding percentages (%), while continuous variables are summarized using median values and interquartile ranges (IQR). To compare categorical variables between groups, the chi-square test or Fisher’s exact test was applied, depending on sample size and expected frequency distribution. Normality of continuous variables was verified using the Shapiro–Wilk test, which indicated non-normal distribution (all *P* < 0.05), prompting the use of the Mann–Whitney *U*-test for group comparisons. A *P*-value of < 0.05 was considered statistically significant, indicating a meaningful difference between the groups. All statistical analyses were performed using IBM SPSS Statistics version 20.0 (SPSS Inc., Chicago, IL, USA).

## Results

### Comparison of general data

Demographic characteristics of the study population are summarized in Table 1. The sample consisted of 101 males (86.3%) and 16 females (13.7%), with a median age of 28 years (IQR, 26–28 years). The majority of participants specialized in digestive surgery (30.8%) or thoracic surgery (20.5%). No significant differences were observed between the two groups in terms of gender or age (all *P* > 0.05).

**Table 1:** Demographic characteristics of the two study groups (*n* = 117)

Variables	Control, *n* (%)	Intervention, *n* (%)	Total, *n* (%)	*P* value
Gender				0.559
Male	53 (84.1)	48 (88.9)	101 (86.3)	
Female	10 (15.9)	6 (11.1)	16 (13.7)	
Age (yrs)				0.871
≤ 28	36 (57.1)	32 (59.3)	68 (58.1)	
> 28	27 (42.9)	22 (40.7)	49 (41.9)	
Specialty				0.950
Digestive surgery	18 (28.6)	18 (33.3)	36 (30.8)	
Thoracic surgery	13 (20.6)	11 (20.4)	24 (20.5)	
Head and neck surgery	10 (15.9)	9 (16.7)	19 (16.2)	
Neurosurgery	10 (15.9)	6 (11.1)	16 (13.7)	
Others^a^	12 (19.0)	10 (18.5)	22 (18.8)	

^a^Including urinary surgery, breast surgery, and bone surgery

### Comparison of communication skills

**Fig. 2:**
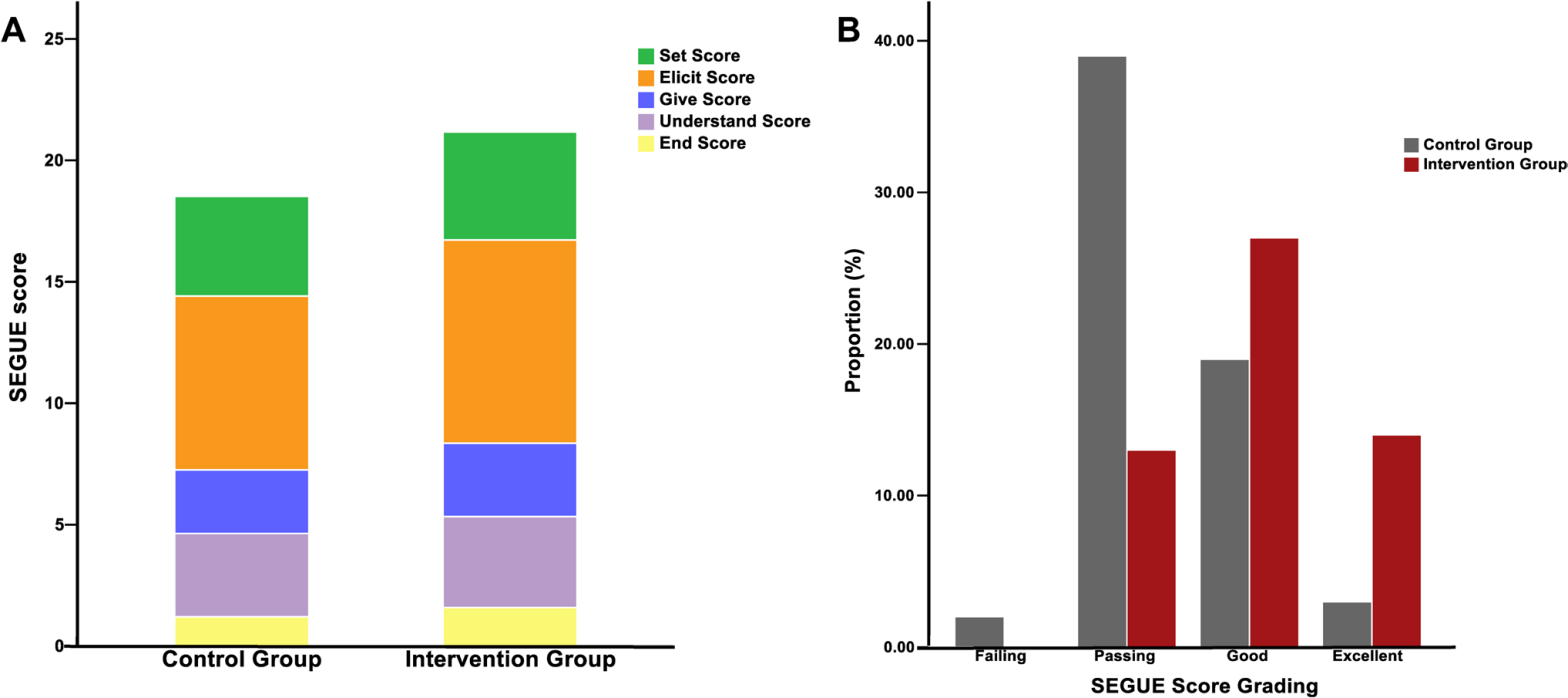
Comparison of the total scores (**A**) and score grading rates (**B**) of SEGUE scores between the two groups

Table 2 shows that the median SEGUE scores for the control and intervention groups were 18 (range, 17–21) and 22 (range, 20–23), respectively. This difference was statistically significant (*P* < 0.001). The intervention group also had higher score rates (defined as the proportion of items with non-zero scores) for individual dimension and total scores of the SEGUE Framework. The control group scored significantly lower on individual SEGUE items (“Set,” “Elicit,” “Give,” “End”) compared to the intervention group (all *P* < 0.05), and more participants in the intervention group achieved excellent total scores (Fig. 2). Table 3 shows that the task, performance, and total scores of the CPPC-AS were significantly higher in the intervention group than in the control group (all *P* < 0.001).

**Table 2:** Comparison of SEGUE scores between the two groups

	Total score	Median (IQR)		*P*^a^ value	Score rate (%)		*P*^b^ value
		Control	Intervention		Control	Intervention	
Set the stage	5	4 (4–5)	5 (4–5)	0.026	81.9	88.9	0.018
Elicit information	10	7 (6–8)	9 (7–9)	< 0.001	71.6	83.7	< 0.001
Give information	4	3 (2–3)	3 (3–4)	0.013	65.5	75.5	0.019
Understand the patient’s perspective	4	4 (3–4)	4 (4–4)	0.120	85.7	93.5	0.006
End the encounter	2	1 (1–2)	2 (1–2)	0.001	60.3	79.6	0.001
Total score	25	18 (17–21)	22 (20–23)	< 0.001	64.4	84.7	< 0.001

^a^Using the Mann–Whitney *U* test

^b^Using the two-sided chi-square test

**Table 3:** Comparison of CPPC-AS scores between the two groups

Grouping	*N*	Task score (total 40 points)	Performance score (total 60 points)	Total score (total 100 points)
Control group	63	34 (33–36)	55 (52–57)	89 (87–91)
Intervention group	54	37 (35–38)	57 (55–58)	94 (90–97)
*Z* value		− 4.664	− 3.771	− 4.785
*P* value		< 0.001	< 0.001	< 0.001

### Comparison of standardized patient satisfaction

**Fig. 3:**
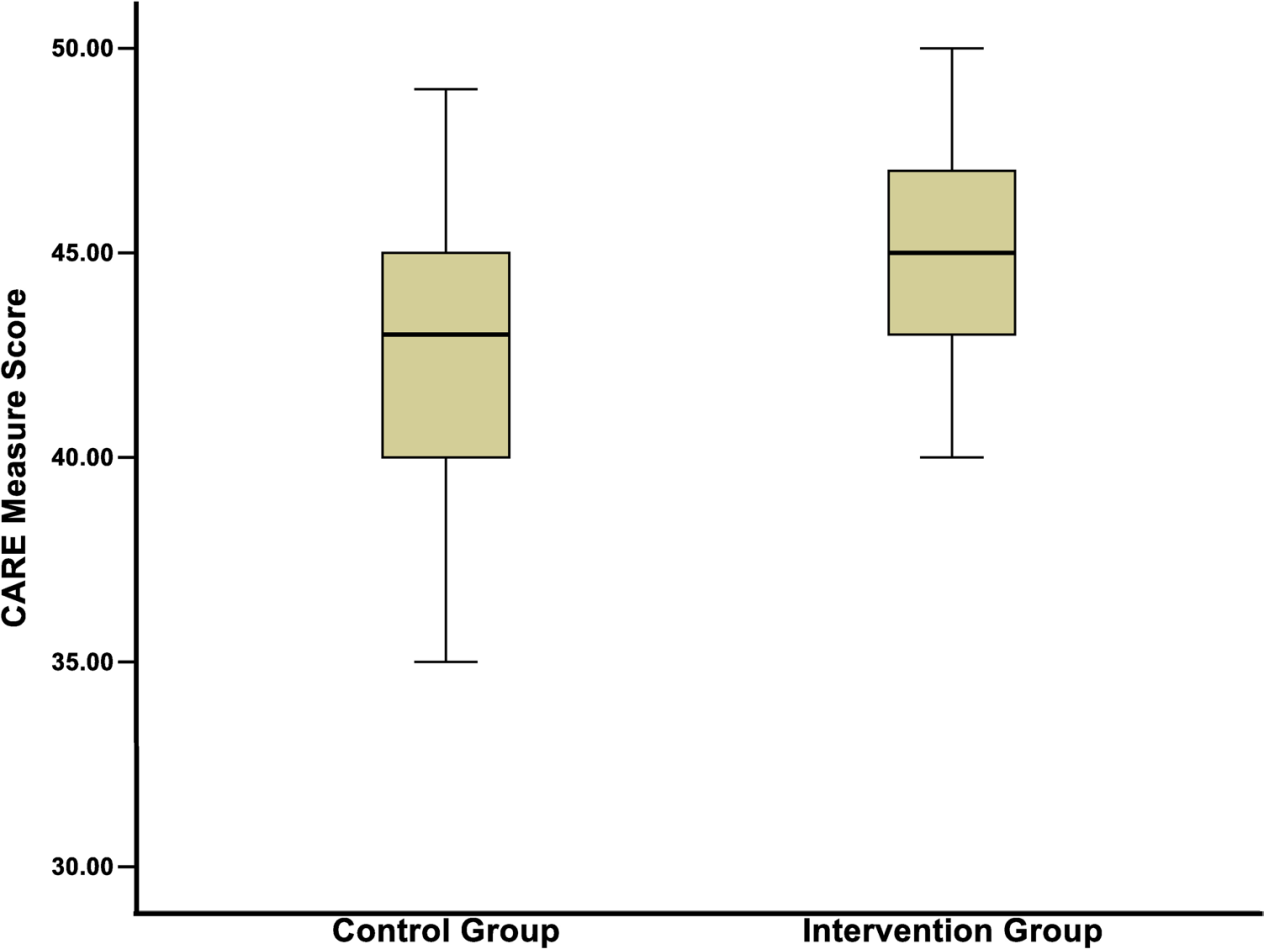
Box plot for CARE measure scores between the two groups

Figure 3 illustrates that the median CARE measure scores for the control and intervention groups were 43 (range, 30–49) and 45 (range, 40–50), respectively, with a significant difference (*P* < 0.001). The intervention group had higher “excellent” rates (defined as a score of 5 points) on items 1, 2, 3, 4, 7, and 8 compared to the control group (all *P* < 0.05). Detailed results are presented in Table 4.

**Table 4:** Comparison of the excellent rates (*n*, %) of CARE measure scores between the two groups

CARE measure item	Excellent rate		*χ*^2^	*P* value
	Control	Intervention		
Making you feel at ease	30 (47.6)	44 (81.5)	5.830	0.016
Letting you tell your “story”	33 (52.4)	38 (70.4)	3.944	0.047
Really listening	34 (54.0)	42 (77.8)	7.241	0.007
Being interested in you as a whole person	26 (41.3)	34 (63.0)	5.477	0.019
Fully understanding your concerns	35 (55.6)	35 (64.8)	1.037	0.308
Being compassionate	36 (57.1)	38 (70.4)	2.189	0.139
Being positive	27 (42.9)	37 (68.5)	7.727	0.005
Explaining things clearly	26 (41.3)	33 (61.1)	4.579	0.032
Helping you to help yourself	29 (46.0)	28 (51.9)	0.394	0.530
Deciding on a treatment plan with you	42 (66.7)	41 (75.9)	1.209	0.271

## Discussion

Effective communication is a core competency for clinicians, crucial in bridging medical practices with patient perceptions. In China, insufficient communication stands out as the most prominent factor behind medical disputes, underscoring its significance [17, 18]. Cancer patients, facing complex conditions and uncertain prognoses, often experience significant psychological distress, which further highlights the critical role of communication. Effective communication helps patients understand their treatment plans, expected outcomes, and potential risks, which can reduce anxiety and improve treatment adherence [19]. Inadequate communication training for oncology providers contributes to stress, decreased job satisfaction, and emotional burnout [20].

Traditional teaching methods, often focusing on theoretical knowledge, overlook the importance of practical skills such as humanistic care [21]. Our study found that traditional approaches resulted in suboptimal communication skills, reflected in low SEGUE scores, incomplete information collection, and poor communication control. These methods, influenced by teacher abilities and student comprehension, lead to inconsistent teaching effectiveness and challenges in developing a coherent teaching process. Students often lack confidence and struggle with complex situations. Therefore, it is essential to explore diverse, practice-oriented teaching models to enhance residents’ practical communication skills [22].

The AIDET framework emphasizes bidirectional communication, focusing on patients’ emotional needs, relationship-building, and decision-making, thereby enhancing healthcare providers’ delivery of information [23, 24]. Consistent with Braverman and colleagues [12], we found that AIDET-based teaching facilitates implementation, with its universal template helping residents overcome timidity and fostering trust among patients. Situational simulation teaching, integrating case analysis, role-playing, dialogue practice, and contextual teaching, enhances residents’ ability to perceive and respond to real-world medical scenarios, including recognizing cultural, linguistic, and time constraints. This targeted, repeatable, and safe method proves effective in teaching communication skills. Our study found that simulation scenarios based on clinical contexts, with contemporary feedback, significantly improved trainee participation, enthusiasm, and initiative, making the teaching process more dynamic.

This study introduced the SST-AIDET model, combining AIDET with situational simulation teaching, which demonstrated distinct advantages. AIDET theoretical teaching helps residents grasp the framework’s principles and applications. The teaching team designed scripts based on daily practice scenarios, recreating challenging cases to improve residents’ adaptability and application of AIDET. Group discussions and teacher feedback identified process issues and facilitated performance improvement. The SST-AIDET group outperformed the control group in SEGUE and CPPC-AS scores, indicating its effectiveness in enhancing communication skills. Due to the role-playing fostering greater empathy, and compassion, the intervention group also showed significantly higher CARE scores, particularly in making patients feel at ease, explaining conditions clearly, and providing positive encouragement. This suggests that SST-AIDET-trained residents are better equipped to listen, understand, and respond to patients, improving healthcare experiences. This is vital for enhancing physician–patient relationships, alleviating patient anxiety, and improving prognoses.

Similar studies combining AIDET or situational simulation teaching with other tools have shown improved teaching effectiveness [25, 26], which aligns with our findings. Future research should explore combined methods that can adapt to various medical environments and patient needs, offering scientifically grounded and effective teaching strategies [27].

This study has several limitations. First, this study tested a small group of students in a hospital for a short period of time, and the lack of random parallel grouping may have introduced biases. In addition, some trainees initially focused too heavily on AIDET steps, leading to rigid performances that lacked fluidity. Additionally, satisfaction of clinical patients and residents was not evaluated, limiting the assessment of teaching effectiveness [28]. The curriculum also included a limited number of simulated scenarios which did not encompass specific oncology specialties. Therefore, future research should expand the sample size, incorporate diverse simulation scenarios and scripts, and assess real patient feedback and treatment outcomes. For example, adapting AIDET to incorporate developmentally appropriate language for pediatric patients or advanced empathy techniques for end-of-life discussions may further refine the SST-AIDET model’s applicability.

## Conclusions

The SST-AIDET teaching model, which integrates the AIDET framework with situational simulation teaching, is highly effective in doctor-patient communication teaching for oncology residents. It effectively enhances their communication skills, promotes learning enthusiasm, and may improve standardized patient satisfaction. This study presents a new approach to communication education, which holds the promise of strengthening physician–patient relationships and improving the quality of medical services.

## Supplementary Information

Below is the link to the electronic supplementary material.

Supplementary file1 (DOCX 18 KB)

Supplementary file2 (DOCX 19 KB)

Supplementary file3 (DOCX 16 KB)

## Data Availability

The authenticity of this study was validated by uploading key raw data to the Research Data Deposit public platform (www.researchdata.org.cn) with RDD number RDDA2024542589.
