# Supplementary material for: Effect of situational simulation teaching combined with the AIDET framework on communication skills training for oncology residents
Source: Support Care Cancer. 2025 May 28;33(6):505. doi: 10.1007/s00520-025-09570-y (PMC12119641; doi:10.1007/s00520-025-09570-y)
Supplement: Supplementary file 1 — Supplementary file1 (DOCX 18 KB) [file 520_2025_9570_MOESM1_ESM.docx]

Table S1. The theoretical teaching of AIDET communication framework.

| Procedures | Interpretations | Illustrations |
| --- | --- | --- |
| Acknowledge | - Greet patients with courtesy, addressing them appropriately based on their age, profession, or any known cultural or personal preferences. Tailor your communication style to meet the needs of vulnerable groups, such as children, elderly patients with cognitive impairments, or those experiencing emotional distress. - Use bed numbers sparingly and only for identification when necessary. - Speak in a gentle tone, use kind and considerate language, and maintain a natural smile to foster a positive doctor-patient relationship. - Be attentive to non-verbal communication by maintaining appropriate eye contact that feels natural and non-intimidating. When suitable and culturally acceptable, express empathy through gentle physical gestures, such as a reassuring pat on the shoulder. | - For a middle-aged patient who appears to be a teacher, you could say: *"Good morning, Mr./Mrs. [Last Name]. It's a pleasure to meet you. I understand you’re a teacher，a truly admirable profession. I’m confident you’ll recover soon and return to inspiring your students. How are you feeling today?"* - For a child patient, try: *"Hi, sweetie! Let's have some fun while we talk about how you're feeling. Would you like that?"* - For an elderly patient with dementia, say: *"Grandpa/Grandma, we’re here to take good care of you. You’re safe with us."* - When speaking to an emotional patient, offer: *"I can see that you’re feeling upset right now. We’re here to help. Let’s take a moment to calm down, and then we can work through this together."* |
| Introduce | - Introduce yourself clearly by stating your title, department, professional competences, and areas of expertise to build trust with patients and their families. - Share examples of successful treatments you’ve managed to instill confidence in the patient’s recovery journey and to encourage open communication and cooperation. - Take a moment to introduce each team member involved in the patient’s care, emphasizing their roles and contributions. | - *"I’m Dr. [Your Name] from the Oncology Department, and I’ll be your attending doctor during your stay. Our team specializes in advanced diagnostics and personalized treatment regimens. For instance, just three months ago, we treated a patient in this very room who was diagnosed with advanced gastric cancer. Through a collaborative effort involving our nurses and specialists in internal medicine, surgery, and radiotherapy, we developed a tailored treatment plan. Today, that patient is cancer-free."* - *"I’m confident that we can provide you with the highest level of care and support. Together, we’ll work towards your recovery. Please feel free to ask any questions—my goal is to ensure you’re comfortable, informed, and supported every step of the way."* |
| Duration | - Clearly explain the next steps in diagnosing and treating the condition, including the required examinations, why they are necessary, and how long each will take. - Provide a detailed timeline of the treatment process, outlining the key activities at each stage, the expected outcomes, and any potential complications. - Discuss any possible discomforts patients may experience during the process and explain the role their cooperation plays. This helps them prepare mentally and reduces anxiety. | - *"We will begin with an abdominal ultrasound to assess your internal organs for any abnormalities. This procedure usually takes about 10 minutes. During the exam, you may feel some pressure on your abdomen. Please try to remain still and follow the technician's instructions."* - *"We may also conduct a blood test to check for tumor markers. This is a quick procedure that takes only a few minutes. You’ll simply need to sit comfortably while the nurse collects a blood sample."* - *"As part of your treatment, following the surgery in the first week, it’s normal to experience some pain at the wound site. We’ll ensure you have appropriate pain relief. By the second week, you’ll be encouraged to begin light activities, such as walking, while avoiding any strenuous exercises."* |
| Explanation | - Explain the key aspects of the disease in clear, simple language, adjusting your communication style based on the patient's cultural background and educational level to ensure understanding. - Present information in a structured and logical sequence, making sure it is relevant and easy to follow. - Be mindful of patients' emotional reactions, especially when delivering difficult news. Give them the time and space to process their emotions, and use empathetic language to convey your understanding and support. - Encourage patients to share their feelings and concerns. This will help you gather specific information and provide explanations that are tailored to their needs. | - *"I know you've been feeling unwell, and we’ve run some tests. The results show that you have [Cancer]. I understand this news may be overwhelming, and I want to assure you that I am here to support you through this. Let’s start by talking about the diagnosis. Think of your body as a complex machine, and right now, we’ve identified [describe the problem in simple terms]. Now, let’s discuss treatment options. We have several approaches available, and we’ll select the one that’s best suited to your situation，and [describe the treatment plan in simple terms]. Finally, we’ll talk about the possible effects after treatment. Does everything make sense so far? How are you feeling about the diagnosis, the treatment, or anything else on your mind?"* - *"I know this is difficult news to hear, and I can’t imagine how you must be feeling right now. Please take all the time you need to process this. I’m here to answer any questions or concerns you may have, whenever you're ready."* |
| Thank You | - At the end of the consultation, sincerely thank the patients and their families for their cooperation, attentiveness, and understanding. - Acknowledge their efforts in actively participating in the discussion and sharing their concerns. - Before concluding, ask if they have any remaining questions or needs, ensuring they feel fully heard and that all their concerns are addressed. - Provide clear information on how they can reach out for further assistance or support after the consultation, including contact numbers or online resources. | - *"Thank you very much for your cooperation, patience, and understanding during today’s consultation. We truly appreciate your willingness to engage and share your thoughts with us. Is there anything else you would like to discuss or any questions you have?"* - *"If you have any concerns or need further information after leaving, please feel free to call our clinic at [phone number] or visit ourpatient portal at [website URL] for additional resources and support."* |
