# Supplementary material for: Effect of situational simulation teaching combined with the AIDET framework on communication skills training for oncology residents
Source: Support Care Cancer. 2025 May 28;33(6):505. doi: 10.1007/s00520-025-09570-y (PMC12119641; doi:10.1007/s00520-025-09570-y)
Supplement: Supplementary file 2 — Supplementary file2 (DOCX 19 KB) [file 520_2025_9570_MOESM2_ESM.docx]

Table S2: Design of Communication Script for Scenario-Based Teaching

| **Visit Process** | Initial Consultation | After Diagnostic Results | Pre-Treatment | Pre-Discharge |
| --- | --- | --- | --- | --- |
| **Location** | Clinic Room | | Hospital Ward | |
| **Task** | Routine Inquiry | Discuss Treatment Plan | Obtain Informed Consent | Instructions |
| **Role Players** | | | | |
| Patient  Family Member | Follows Script with Emotions and Responses | | | |
| Doctor | Guided by AIDET Framework | | | |
| **AIDET Framework—Taking the obtaining informed consent before surgery as an example** | | | | |
| **Acknowledge** | - **Doctor:** *"Hello, and thank you for placing your trust in our team. We’re here to support you throughout this challenging process. How are you feeling about the upcoming surgery?"* - **Patient** (Apprehensive): *"I’m scared, doctor. I’ve heard so many things about this procedure. Will I be okay?"* - **Doctor** (Reassuringly): *"It’s completely normal to feel afraid. We’re here to address all your concerns and ensure you’re as prepared as possible for the surgery."* | | | |
| **Introduce** | - **Doctor:** *"Before we begin, I want to make sure you fully understand the details of your upcoming surgery. I’m Dr. [Name], and I will be overseeing your case. The surgical team/radiation oncology team is highly experienced and skilled in performing this procedure."* - **Patient/Family Member:** *"Doctor, can you tell me more about the team? I want to know who will be taking care of me."* - **Doctor:** *"Certainly. The surgeon, Dr. [Surgeon’s Name], has successfully performed many procedures like this one. The anesthesiologist, Dr. [Anesthesiologist’s Name], will ensure your comfort and safety throughout the procedure. Additionally, our nursing staff will be with you every step of the way during your recovery."* | | | |
| **Duration** | - **Doctor:** *"This conversation will take about 20 minutes, but we’ll take as much time as necessary to make sure you understand everything. First, I’ll explain the procedure in detail, which will take around 10 minutes. Then, we’ll discuss the pre-operative/pre-treatment preparations, which will take another 10 minutes. Please feel free to ask questions at any time."* - **Patient/Family Member:** *"Thank you, doctor. I have a lot of questions, especially about the recovery."* - **Doctor:** *"That’s completely understandable. We’ll address all of your questions, including those about recovery and more."* | | | |
| **Explanation** | - **Doctor:** *"The surgery/treatment involves [procedure]. For the surgery, you’ll be under anesthesia, and the surgeon will make an incision [describe the location and size]. The goal is to remove the tumor as completely as possible while minimizing damage to surrounding tissues. We’ll closely monitor your vital signs during the procedure to ensure your safety. Afterward, you’ll wake up in the recovery room, where you’ll be observed for a few hours. You may feel some pain and discomfort, which we will manage with appropriate medications. If you're receiving chemotherapy, you’ll either take the drugs through an IV or orally, depending on the specific regimen. Treatment is given in cycles, with rest periods in between to help you recover. You may experience side effects such as nausea, fatigue, and hair loss, but we have effective ways to manage them and ensure your comfort. To prepare for the surgery/treatment, you’ll need to stop eating and drinking [specify time before procedure], and bring personal items such as comfortable clothes and toiletries."* - **Patient/Family Member:** *"What about the risks? I’m really worried about something going wrong."* - **Doctor:** *"Every medical procedure carries some risk, but we take every possible precaution to minimize them. Our surgical team is highly trained and experienced, and we have a comprehensive plan in place to address any potential complications. For chemotherapy, we’ll closely monitor your blood counts and other health indicators to ensure your safety. I’ll go over the risks in more detail, but please know that we’ll be with you every step of the way to address any concerns that may arise."* - **Family Member:** *"Doctor, how long will the recovery take? We need to plan for that."* - **Doctor:** *"Recovery time varies depending on the individual and the extent of the surgery/treatment. For surgery, you may stay in the hospital for [X] days, and it could take a few weeks to a few months to fully recover. After chemotherapy, you may experience fatigue after each cycle, but your energy levels should gradually improve. We will provide you with detailed instructions on how to care for yourself during the recovery period."* | | | |
| **Thank You** | - **Doctor:** *"Thank you for your trust. We are fully committed to your care and well-being. The entire team is dedicated to making this process as smooth as possible for you. If you have any last-minute questions or concerns before the procedure, please don’t hesitate to ask. We’ll see you soon."* - **Patient:** *"Thank you, doctor. I’m still scared, but I feel a bit more confident knowing that you and the team are taking care of me."* - **Family Member:** *"Thank you, doctor. We’ll be praying for a successful outcome."* | | | |
