# Supplementary material for: Effect of situational simulation teaching combined with the AIDET framework on communication skills training for oncology residents
Source: Support Care Cancer. 2025 May 28;33(6):505. doi: 10.1007/s00520-025-09570-y (PMC12119641; doi:10.1007/s00520-025-09570-y)
Supplement: Supplementary file 3 — Supplementary file3 (DOCX 16 KB) [file 520_2025_9570_MOESM3_ESM.docx]

Table S3. Chinese Physician-Patient Communication Assessment Scale

| **TASK items** | poor | fair | good | very good | excellent | Score |
| --- | --- | --- | --- | --- | --- | --- |
|  | 1 | 2 | 3 | 4 | 5 |  |
| 1.Self-introduction and clarification of the purpose of the conversation. |  |  |  |  |  |  |
| 2. Confirm the patient's family members and inquire if others will be attending. |  |  |  |  |  |  |
| 3. Provide a detailed explanation of the patient's current condition, diagnosis, and the next steps in treatment. |  |  |  |  |  |  |
| 4. Discuss the surgical plan in detail, including associated risks. |  |  |  |  |  |  |
| 5. Respond appropriately to any questions from the family about the patient's condition. |  |  |  |  |  |  |
| 6. Address any questions from the family regarding the surgery and treatment plan. |  |  |  |  |  |  |
| 7. Respond appropriately to other questions from the family. |  |  |  |  |  |  |
| 8. Provide a summary and confirmation of key points discussed. |  |  |  |  |  |  |
| **Performance Items** | poor | fair | good | very good | excellent | Score |
|  | 2 | 3 | 4 | 5 | 6 |  |
| 1. Clear and logical communication of information. |  |  |  |  |  |  |
| 2. Demonstration of empathy and compassion. |  |  |  |  |  |  |
| 3. Providing humanistic care (allowing time to address emotional fluctuations of family members and offering comfort). |  |  |  |  |  |  |
| 4. Collaborating with the family to develop a treatment pla |  |  |  |  |  |  |
| 5. Avoiding the use of overly complex or technical language. |  |  |  |  |  |  |
| 6. Using language consistent with the patient's actual situation. |  |  |  |  |  |  |
| 7.Ensuring communication is based on equality |  |  |  |  |  |  |
| 8. Committing to providing the patient with the best possible effort and support. |  |  |  |  |  |  |
| 9. Maintaining a comfortable pace, tone, and clarity of speech. |  |  |  |  |  |  |
| 10. Making appropriate eye contact. |  |  |  |  |  |  |
| **Total Score** |  |  |  |  |  |  |

It was rated on a 5-point Likert scale ranging from 1 to 5 (1 = poor, 2 = fair, 3 = good, 4 = very good, 5 = excellent).
